# Effects of slaughtering operations on carcass contamination in an Irish pork production plant

**DOI:** 10.1186/2046-0481-67-1

**Published:** 2014-01-18

**Authors:** Paul Wheatley, Efstathios S Giotis, Aideen I McKevitt

**Affiliations:** 1 NICHE, School of Biomedical Science, University of Ulster, Coleraine, UK; 2 School of Agriculture and Food Science, University College Dublin, Belfield, Dublin 4, Ireland

**Keywords:** TVC, *Enterobacteriaceae*, HACCP, Critical control points, Pork slaughtering

## Abstract

**Background:**

Microbiological standards within pork slaughter processing plants in the European Union are currently governed by Commission Regulation (EC) 2073/2005, which describes detailed performance criteria at specific stages of the procedure (following carcass dressing and before chilling) for total viable counts (TVC), *Enterobacteriaceae* (EB) and *Salmonella* spp. In this study, 95 carcasses from an Irish pork slaughter plant were sampled by swabbing 100 cm^2^ of surface at three sites (belly, ham, jowl) to examine the effects of eight processing stages (stunning, bleeding, scalding, singeing, polishing, evisceration, final inspection and chilling) on contamination levels.

**Results:**

TVC ranged from approximately 1.7–6.3 log cfu cm^2^ during sampling. There were significant reductions in TVC for all sites after scalding and singeing (*p* < 0.05), whilst there was a significant increase in counts after polishing and evisceration (*p* < 0.05) compared with preceding stages. EB counts indicated hygienic weak points in the examined slaughter plant leading to faecal (cross)-contamination, with elevated counts after stunning, bleeding and evisceration (*p* < 0.05), compared with final counts after chilling.

**Conclusions:**

Although the bacterial numbers reported in this study may reflect specific plant practices and temporal influences, results show that contamination can be introduced at various steps in the process and highlight the importance of monitoring locations other than those required by legislation within the process. Monitoring can be used to establish baseline levels for high-risk stages specific to each plant and to assess the effectiveness of additional interventions.

## Background

Recognizing an increased number of food safety problems associated with pork consumption such as *Salmonella* outbreaks and taking into account rising consumer concerns, the European Union established strict microbiological criteria for pork slaughtering operations. Microbiological standards within pork slaughter processing plants within the European Union are currently governed by Commission Regulation (EC) 2073/2005. The regulation dictates that safety in pork processing should be ensured principally by preventive approaches, such as the implementation of good hygiene practices and the application of risk management procedures based on HACCP (hazard analysis and critical control points) principles [1].

The regulation designates specific process hygiene criteria for total viable counts (TVC) and *Enterobacteriaceae* for the post-evisceration and pre-chilling stages, which provide useful data for the validation and verification of HACCP procedures and other hygiene control measures employed in the pork industry [2-5]. Hence, testing of carcasses for TVC provides an effective assessment of overall hygiene conditions in facilities where pigs are slaughtered and processed. Results are categorised as satisfactory, acceptable, and unacceptable, when TVC counts fall within the following ranges <4.0, 4.0-5.0, and >5.0 cm^2^ respectively. *Enterobacteriaceae* are also a useful measure of hygienic performance, indicating probable faecal contamination with mean log counts of <2.0, 2.0-3.0, and >3.0 cm^2^ stipulated in the legislation for satisfactory, acceptable, and unacceptable categories respectively.

Since the implementation of HACCP-based food safety management systems, additional challenges are posed to smaller scale abattoirs, where full execution of more elaborate systems may not be feasible. Evidence has shown that standards of slaughter hygiene can vary between abattoirs as a result of controls being implemented inadequately or inconsistently at key processing stages. Such deficiencies can lead to breaches in hygiene and resultant carcass contamination [2,6,7]. Given the lack of targeted studies and adequate summary statistics, it is not possible to infer with confidence if HACCP principles are rigorously implemented in all pork slaughterhouses and consequently propose corrective actions to improve the efficiency of current legislation and slaughterhouse practices**.**

Therefore, more primary research and access to slaughterhouse microbiological data is needed to evaluate properly HACCP program effectiveness. The aim of this study was to obtain data on the microbiological contamination of pig carcasses in a medium-scale Irish abattoir at the various stages of the process, and evaluate the need for improvement of monitoring in pork slaughter facilities.

## Methods

### Slaughter plant and processing

This study was carried out in a medium-scale, HACCP certified, Irish pork production plant processing a rate of approximately 1,200 pigs per day. Following transportation, pigs were unloaded and placed in designated, marked pens in such a way as to minimize stress. All animals underwent ante-mortem inspection in the lairage prior to being stunned using CO_2_ (Monicon CO Detector, Ireland). Stunned animals were bled by cutting their carotid arteries using a sterile knife. During the scalding phase, pigs were submerged into the scalding tank filled with 60°C tap water, for 5–10 minutes (Bitterling, UK). Carcasses were de-haired by rotating and then singed using a gas flame for 25 seconds (Bitterling, UK). Polishing was conducted using a cold-water spray and rubber flails rotating and moving in opposing directions. Carcasses were transported into the clean dressing area and prepared for bunging/evisceration which involved slitting open the belly cavity, bagging the bung sufficiently to prevent leakage and removing the gastrointestinal tract. The pluck, liver and tongue were then removed before carcass splitting using a saw (Kentmaster, USA) and subsequent spinal cord removal. Prior to final inspection the lard, kidneys and diaphragm were removed. After inspection carcasses underwent further dressing and trimming in order to remove any visible marks and blood clots. Cold tap water was used to wash the carcass and washed carcasses were chilled to 2-4°C overnight. According to the facility’s HACCP plan random microbial tests are performed before and after singeing, evisceration and chilling, records are kept and reviewed daily and the procedure is verified by monthly audits.

### Sampling and enumeration of microorganisms on carcasses

A total of 95 randomly selected carcasses, were sampled at three sites (ham, belly and jowl) after eight key processing stages (stunning, bleeding, scalding, singeing, polishing, evisceration, final inspection and chilling). Sampling was completed over 19 visits to the plant, and processing of samples from 5 carcasses (after each visit) was done within an hour after collection. Sterile polyurethane sponge swabs moistened with 10 ml maximum recovery diluent (MRD, Oxoid) in sterile stomacher bags, were used to swab areas of 100 cm^2^ from the same carcass site at each processing stage. After swabbing each sponge was replaced into the sterile bag and stomached individually using a peristaltic stomacher working at a speed of 250 cycles/min for 2 minutes using 100 ml of sterile MRD. Serial decimal dilutions of the resultant suspension were prepared in MRD, plated on Plate Count Agar (PCA, Oxoid) for TVCs and Crystal Violet Neutral Red Bile Lactose Agar (VRBL, Oxoid) for *Enterobacteriaceae* (EB), and aerobically incubated at 30 ± 1°C for 72 hours and 37 ± 1°C for 24 hours, respectively. After incubation colonies were counted for each plate.

### Statistical analyses

The resultant counts for TVC and EB at each of the jowl, belly and ham at each processing stage were transformed into log cfu/cm^2^ values and the effect of each processing stage was assessed by two-way Anova using SPSS Version 17.0 (SPSS Inc. Chicago). Posthoc multiple comparisons with a Tukey’s test allowed comparisons of bacterial counts between sampling sites and processing stages. Mean differences were considered significant when *p* < 0.05.

## Results

### Total viable counts

**Figure 1:**
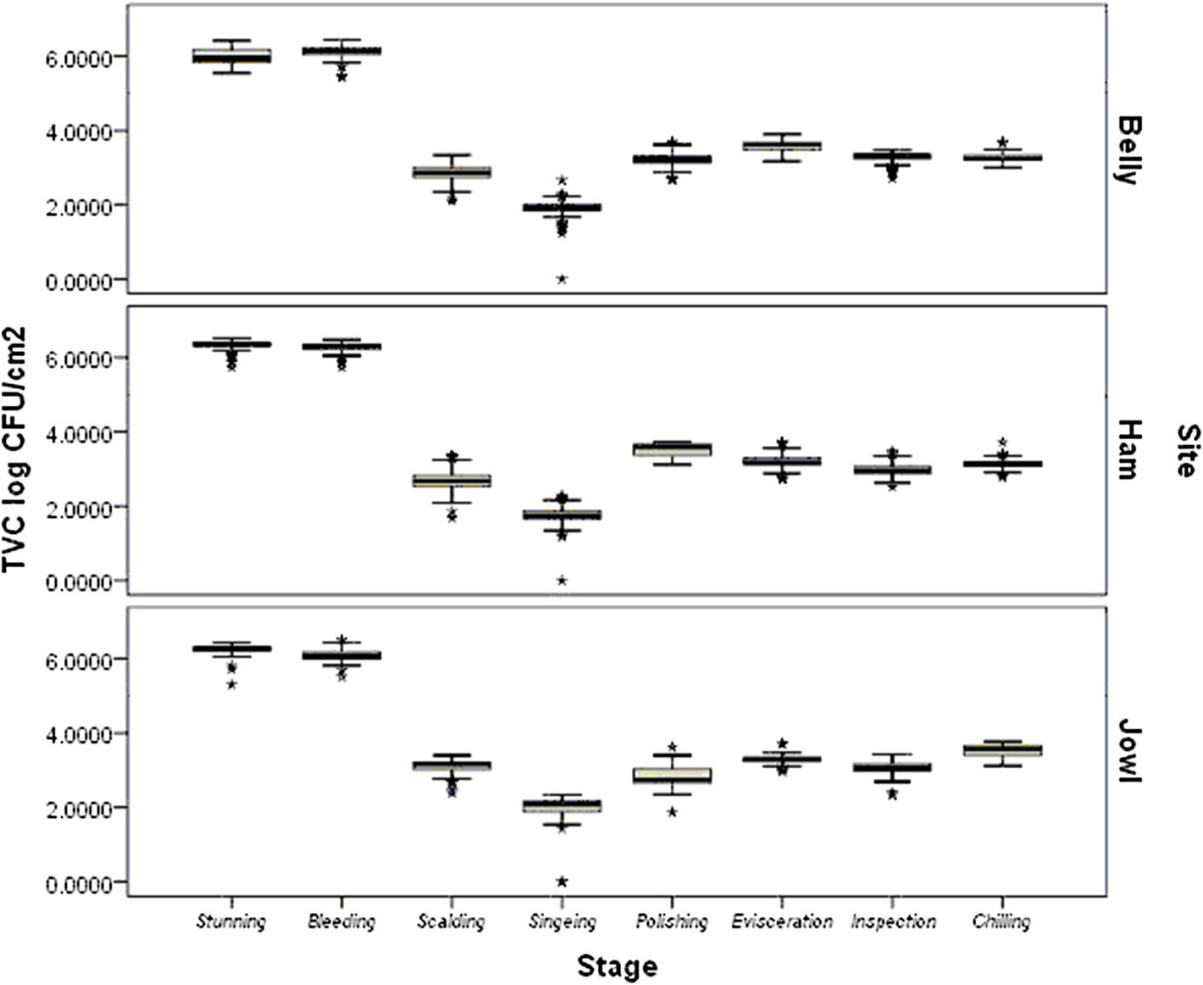
**TVC counts (log cfu/cm**^**2**^**) on jowl, belly and ham at different stages of the slaughter process.** Broken line indicates TVC limit according to the performance criteria of Regulation (EC) No 2073/2005.

The compiled logarithmic data for total viable counts (TVC) from samples of belly, ham and jowl after eight stages of carcass processing are presented in Figure 1. For a more comprehensive interpretation, results are presented in box plots; the box contains the middle 50% of the data, the upper edge (hinge) of the data indicates the 75^th^ percentile and the lower hinge the 25^th^ percentile of the dataset distribution. TVC ranged from approximately 1.7 - 6.3 log CFU/cm^2^ during sampling. There were significant reductions in TVC for all sampling sites after scalding and singeing (*p* < 0.05) while there was a significant increase in counts after polishing and evisceration (*p* < 0.05).

The average TVCs from ham, belly and jowl after stunning were 5.98, 6.33, and 6.25 log CFU/cm^2^ and after bleeding 6.11, 6.26 and 6.07 log CFU/cm^2^ respectively. After scalding, TVCs from all three sites were reduced significantly (*p* < 0.05) to approximately 2.8 log CFU/cm^2^. After singeing, TVCs for all sites were further reduced to approximately 1.8 log CFU/cm^2^ (*p* < 0.05). TVCs for all sites increased significantly (*p* < 0.05) after polishing. Results for ham showed TVC decreasing from 3.52 after polishing, to 3.19 log cfu/cm^2^ after evisceration, further reduced to 2.97 after final inspection and increased to 3.14 log cfu/cm^2^ after chilling. TVCs for belly and jowl increased following final inspection and counts for jowl further increased (*p* < 0.05) after chilling, by approximately 0.5 log cfu/cm^2^ compared to previous sampling.

### Enterobacteriaceae

**Figure 2:**
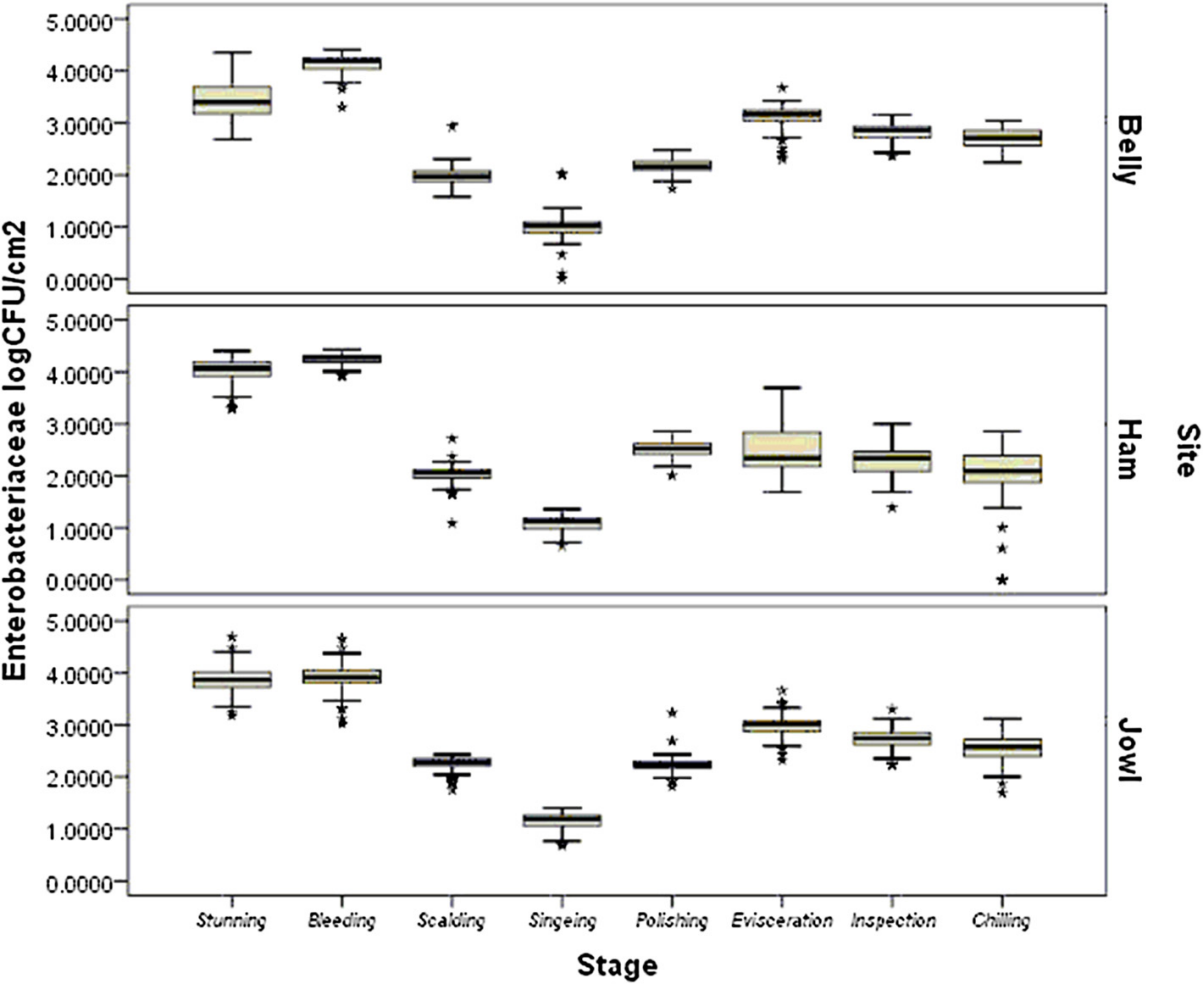
**Mean *Enterobacteriaceae *counts (log cfu/cm**^**2**^**) on jowl, belly and ham at different stages of the slaughter process.** Broken line indicates *Enterobacteriaceae* limit according to the performance criteria of Regulation (EC) No 2073/2005.

The levels of *Enterobacteriaceae* (EB) for belly, ham and jowl are presented in Figure 2. EB were statistically different after each processing stage (*p* < 0.05) while less significant was the difference between counts following final inspection and chilling (*p* < 0.05). Sampling site had also a significant effect in the EB numbers observed (*p* < 0.05).

EB counts in ham, jowl and belly samples were 4.1, 3.81 and 3.25 logcfu/cm^2^ after stunning. Numbers increased significantly after bleeding for all three sites by increments of 0.16, 0.13 and 0.96 log cfu/cm^2^ (*p* < 0.05), respectively. There was a significant reduction after scalding and singeing at all three sites (*p* < 0.05). Ham swabs showed the most significant decrease in counts with a mean reduction of 2.96 log cfu/cm^2^ from stunning to singeing. Numbers subsequently increased significantly (*p* < 0.05) after polishing for all three sites. After evisceration, the jowl and belly samples showed a significant increase in EB counts (*p* < 0.05), however counts from ham swabs decreased from 2.54 to 2.25 log cfu/cm^2^. This was followed by a further reduction (*p* < 0.05) after final inspection and the final levels on carcasses after chilling for jowl, belly and ham were 2.42, 2.68 and 2.04 logcfu/cm^2^ respectively.

## Discussion

It is widely believed that the EU microbiological criteria for carcasses may not reflect all operating conditions in a pig slaughtering procedure, and should be seen as baseline [8]. However, regular monitoring of process hygiene in plants is an essential verification procedure within HACCP-based food safety management systems to ensure microbiological contamination is effectively controlled. EU microbiological criteria are designed to assess hygiene only after carcass dressing and prior to chilling. There are very few studies that have examined microbiological levels at all stages of a pork slaughtering procedure and building up baseline data for each of the process stages could allow for non-legally binding thresholds to be established that can be subsequently used for internal validation of HACCP plans and assessment of hygiene interventions. Baseline data can also be used as a tool to assess the hygienic status of the plant and predict contamination levels that can surpass the legal criteria at the designated sampling points (post evisceration and pre-chilling stages).

The facility’s HACCP plan designates as CCPs the final inspection and chilling steps of the procedure since these are the last steps where visible faecal contamination is removed and temperature is brought down to a safe level to inhibit the growth of microflora respectively.

The majority of TVCs in this study were relatively low when compared with counts at the initial stages of the process (stunning and bleeding), while EB counts provided indications of hygienic weak points in the examined slaughter plant leading to faecal contamination, not evident from TVC data, with counts that exceeded moderate levels after stunning, bleeding and evisceration. Bacterial numbers reported in this study possibly reflect specific practices and temporal influences characteristic of the assessed plant, highlighting the need for monitoring of all process stages in order to identify weaknesses in the implementation of the HACCP management system in place.

TVC results particularly at the initial stages of the process after stunning and bleeding, differ significantly from published data reporting mean pig carcass TVCs less than 5.0 log cfu cm^2^[5-7,9-13]. Ineffective cleaning and lack of decontamination procedures during animal transportation and lairage, may account for the high TVC and EB counts (TVC > 6.0 log cfu cm^2^ and EB > 3.0 log cfu cm^2^), observed after stunning. Heavily contaminated incoming animals in the slaughter facility, due to lack of on-farm or en route hygiene measures, can have a profound effect on microbial levels and pose considerable risks to product quality [2,6,14]. Cross-contamination shortly after bleeding may be further exacerbated by contact of carcasses that may fall from their shackles, with floor contamination and/or wet floor surfaces [8,15].

In contrast, the present study confirmed that scalding and singeing significantly reduced numbers of bacteria (both TVC and EB) in line with previous studies [6,9,10,16]. Both stages are widely considered as CCPs within HACCP systems, but not in the studied facility [7,16]. The decrease in bacterial numbers following singeing is alternated by a significant increase in microbial counts at downstream processing steps, denoting that monitoring should not be limited to specific production stages. A successful HACCP system requires verification/monitoring systems for CCPs and establishment of critical limits. Previously, it has been suggested that each pig slaughter plant should establish its own baseline data for determined CCPs [7]; however, such practices are complicated due to a lack of consensus among researchers and operators on definite pig slaughter line CCPs. Nonetheless, establishment of baseline levels for each processing stage of the examined slaughter plant could have facilitated the identification of specific deficiencies of the facility’s HACCP plan.

After polishing, increased counts were observed for both TVCs and EB. No monitoring procedures and corrective actions were described in the facility’s HACCP plan for this production stage and it is therefore difficult to identify the cause of increased bacterial levels. They may be related to difficulties in sanitising the polishing equipment during use resulting in cross-contamination [8,16,17], or possibly recurring hygienic errors by personnel. Previous studies have reported the build up of contamination on the polishing equipment with increased carcass contamination correlated to longer duration of machine operation. These findings demonstrate the need for regular cleaning of polisher equipment during daily work shifts [18,19].

Evisceration is frequently reported as a major source of contamination of pork carcasses, and this study found significant increases in EB in all three-sample types (p < 0.05). The main risk of carcass contamination during evisceration is the direct or indirect spillage of faecal material from rupture of the gut [20,21]. Other contamination risks occur during the removal of the pharynx, tonsils and tongue that may also be heavily contaminated. Many methods have already been described that reduce the spread of contamination, including tying and sealing the rectum [22], using a two-knife system sanitized at 82°C [6], and using hot pasteurized water prior to evisceration [23]. Despite previous reports describing increases in levels of enteric bacteria during evisceration, some authors suggest that efficiency of evisceration is better controlled by corrective actions and appropriate training of personnel according to optimum Standard Operating Procedures and Good Manufacturing Practice rather than establishing this step as a CCP [2,6,24].

After final inspection and chilling (both CCPs in this facility), TVC levels were relatively low, while EB levels were relatively higher (2.0-3.0 log cfu cm^2^) compared with previous stages. Belly and jowl EB counts were near or over 3.0 log cfu cm^2^ (Figure 2). Final inspection includes examination, excision and palpation of the carcass, intestines and pluck, to detect possible human health risks [25]. Shortly after dressing, and before chilling the numbers as well as the type of microflora found on carcasses reflect the contamination that occurred during the slaughter processing line. Microbiological sampling at this stage is a good indicator of hygiene errors during the operation as well as the microbial load of the slaughtered animal. Chilling modulates levels and composition of carcass bacterial numbers and flora and is gradually dominated by predominantly psychotrophic microorganisms during extended storage. In the present study, chilling resulted in an increase of TVC in jowl (*p* < 0.05) and less significantly in ham, while belly counts were stable, possibly as this part of carcass comes into less contact with other carcasses, the floor and the walls of the chiller. TVC increases were independent of EB populations that were significantly reduced (*p* < 0.05). It is possible that the increase in jowl counts could be explained due to the proximity of the jowl to the floor with water splashing being a contributing factor as previously reported [6,7,9]. Microbial populations found at end-of-slaughter line and end-of-chilling are indicators of different hygiene processes and may not be directly comparable or equivalent [26]. Further microbial contamination of carcasses on their way from inspection point to and during chilling may be due to cross-contamination from workers’ hands, other carcasses, liquid leakage and from aerosols [27-29].

## Conclusions

This study has shown relatively high numbers of EB at several stages of a pork slaughtering process. Singeing resulted in significant reductions of both TVC and EB; however this trend was offset by re-/cross- contamination in later stages of processing. Although TVC results at the end of the pork production process largely fell within EU limits, the overall results provide evidence that monitoring single predetermined CCPs in isolation from the rest of the procedure can mask possible deficiencies of a HACCP plan and thus, there is scope in establishing baseline criteria for each consecutive stage of the production. The study demonstrates the usefulness of monitoring more than one location within the process for each plant so that high risk stages can be identified, increased controls implemented and ongoing monitoring carried out to assess the effectiveness of additional interventions.

## Abbreviations

HACCP: Hazard analysis and critical control point; CCP: Critical control point; EU: European Union; MRD: Maximum recovery diluent.

## Competing interests

The authors declare that they have no competing interests.

## Authors’ contributions

PW carried out the experimental work and drafted the manuscript. EG reviewed the scientific work, helped to draft the manuscript, and conducted the statistical analysis. AM supervised the experimental work and reviewed the manuscript. All authors read and approved the final manuscript.
